# MSICKB: A Curated Knowledgebase for Exploring Molecular Heterogeneity and Biomarker Prioritization in Microsatellite Instability Cancers

**DOI:** 10.34133/csbj.0047

**Published:** 2026-04-20

**Authors:** Yuxin Zhang, Xiaoyu Li, Xin Zheng, Cheng Bi, Jie Song, Zhichuan Xu, Dan Cao, Marcos Gestal Pose, Bairong Shen

**Affiliations:** ^1^ Department of Medical Oncology, Institutes for Systems Genetics, Frontiers Science Center for Disease-related Molecular Network, West China Hospital, Sichuan University, Chengdu, Sichuan 610041, China.; ^2^ Department of Computer Science and Information Technology, University of A Coruña, 15071 A Coruña, Spain.

## Abstract

Microsatellite instability (MSI) is a clinically actionable molecular phenotype in cancer, but MSI-associated findings remain fragmented across tumor types, study designs, and biomarker categories, limiting systematic cross-cancer comparison and evidence-guided biomarker prioritization. To address this problem, we developed the Microsatellite Instability Cancer Knowledgebase (MSICKB), a manually curated and literature-traceable resource for MSI-associated molecular and clinical features. We collected and curated 1,382 MSI-related features from 492 publications covering 31 cancer types and organized the evidence into 4 major dimensions: genetic and molecular alterations, clinicopathological features, prognostic factors, and therapeutic response. Based on curated gene–cancer associations, we constructed a simple bipartite network to examine the cross-cancer organization of MSI-associated genes. In the primary network, 99 genes were linked to 13 cancer types through 147 unique gene–cancer edges. Gene degree was strongly right-skewed, with most genes linked to a single cancer type and a small subset showing broader cross-cancer connectivity. Using an operational cutoff of degree ≥ 3, we identified 9 hub genes: BRAF, CD274, KRAS, MLH1, MSH2, PTEN, RNF43, TGFBR2, and TP53. These hubs were enriched in canonical MSI-related pathways, including mismatch repair, cancer signaling, and immune regulation. To provide external molecular support, we further evaluated the hub genes in 3 The Cancer Genome Atlas cohorts with established MSI relevance. In pooled analyses of 336 MSI-high and 1,214 non-MSI-high tumors, all 9 hub genes showed significant differences in mutation prevalence and expression. Overall, MSICKB provides a structured framework for MSI-related evidence synthesis, cross-cancer comparison, and biomarker prioritization and is freely available at http://www.sysbio.org.cn/MSICKB/.

## Highlights

- Curated MSI knowledgebase spanning 31 cancer types.
- MSICKB integrates 1,382 molecular and clinical features.
- A simple gene–cancer network identified 9 cross-cancer hub genes.
- Hub genes converged on mismatch repair and immune-related pathways.
- MSICKB supports cross-cancer comparison and biomarker prioritization.

## Graphical Abstract

**Figure:**
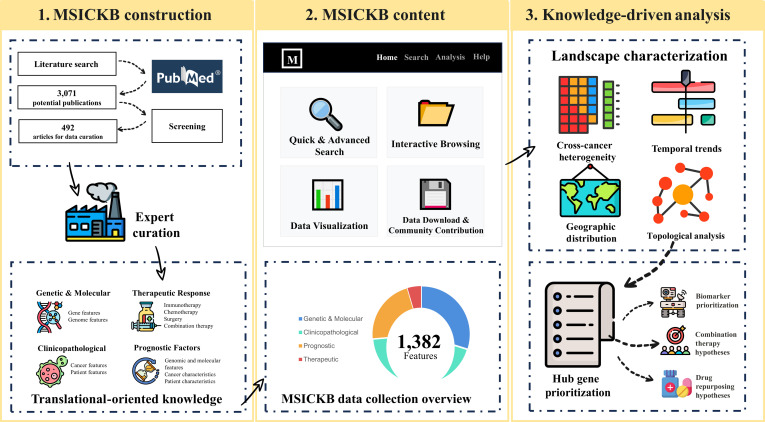

## Introduction

Genomic instability, a hallmark of cancer [1], can manifest prominently as microsatellite instability (MSI) [2]. As a hypermutable phenotype resulting from a defective DNA mismatch repair system [3], MSI is a clinically actionable pan-cancer biomarker for diagnosis (e.g., Lynch syndrome), prognostic stratification, and therapeutic decision-making, most notably for immune checkpoint inhibition [4–9]. However, substantial heterogeneity in clinical outcomes persists even among MSI-high (MSI-H) tumors, suggesting additional molecular subtypes and regulatory programs that remain incompletely characterized [10–14].

A systematic synthesis of molecular and clinical features that differ between MSI-H and microsatellite-stable tumors across cancer types is needed to better characterize this heterogeneity. Although large-scale genomics resources provide broad pan-cancer molecular profiles and mutation-centered annotations, they are not designed to curate literature-reported, MSI-contextualized feature-level associations, particularly those linking molecular markers to clinicopathological characteristics, prognosis, and treatment response, into a standardized and traceable form [15,16].

We therefore developed the Microsatellite Instability Cancer Knowledgebase (MSICKB), a curated, literature-based resource [17] that organizes MSI-associated evidence across cancers with explicit study context and direct traceability to the source publications. MSICKB integrates 4 complementary evidence dimensions—genetic and molecular alterations, clinicopathological features, prognostic factors, and therapeutic response—thereby enabling structured cross-cancer comparison and targeted retrieval of MSI-associated evidence.

We further sought to use the curated resource as a basis for systems-level characterization of the reported MSI landscape and for evidence-guided prioritization of MSI-associated genes across cancers. MSICKB therefore serves as both a reference resource and a basis for evidence synthesis, biomarker-oriented research prioritization, and future integrative studies in MSI-associated cancers [18].

## Materials and Methods

### Data acquisition and curation

#### Search strategy and selection criteria

**Fig. 1.**
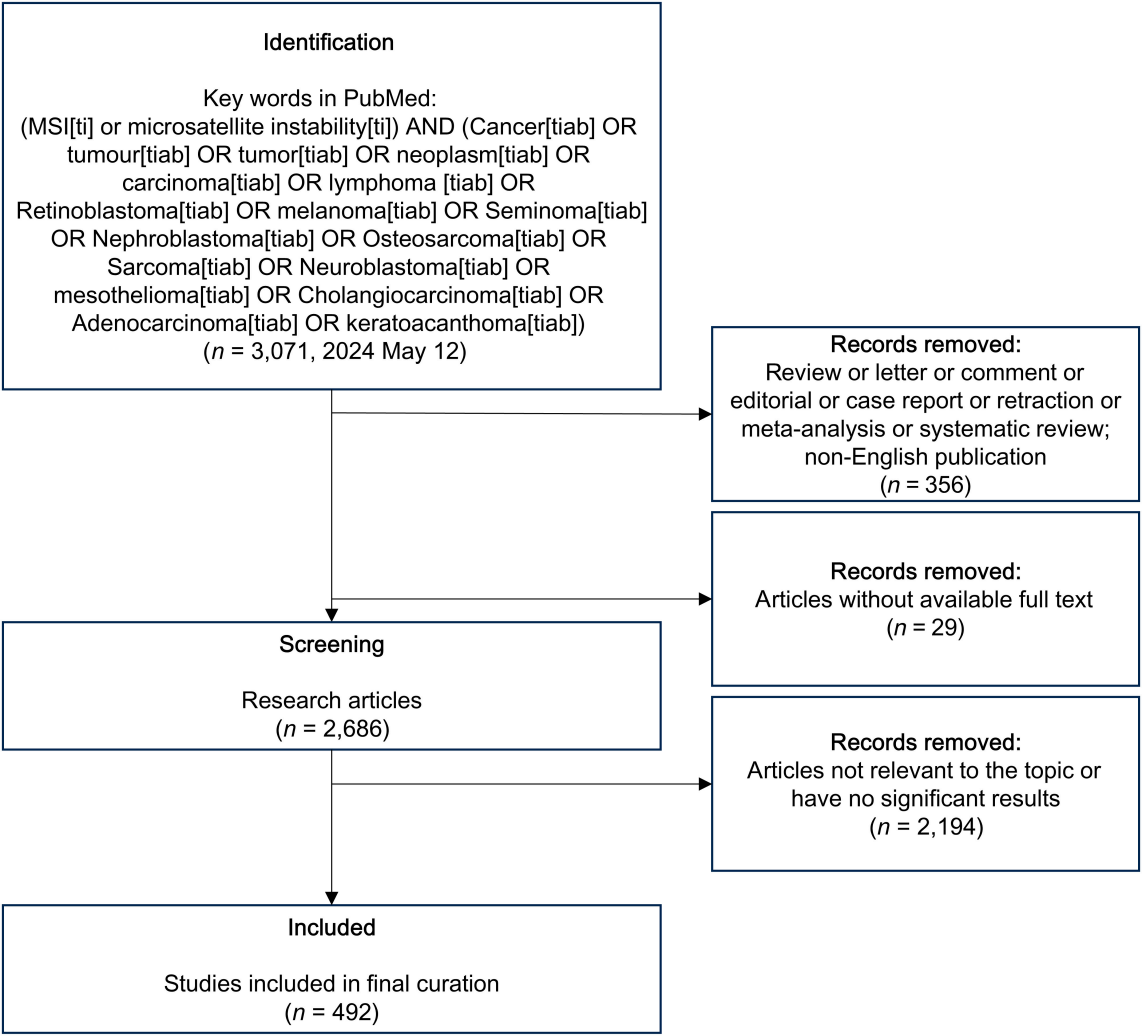
Flowchart of the literature screening and selection process. The diagram illustrates the multistage process for identifying relevant studies for inclusion in the MSICKB. Starting from an initial PubMed search, records were systematically screened with stringent filtering criteria based on publication type, language, full-text availability, and relevance, resulting in a final cohort of 492 articles for data curation.

To construct a comprehensive knowledgebase of features related to MSI in cancer, we performed a systematic literature search of the PubMed database. Our search strategy prioritized sensitivity, employing a free-text search within the title and abstract fields to capture relevant studies that might not be consistently indexed with MeSH terms. Keywords and queries related to MSI and specific cancer types, executed up to 2024 May 12, were used for the search strategy (detailed search strings are provided in Fig. 1).

This initial search yielded 3,071 potentially relevant publications. Figure 1 shows the multistage screening process we applied. First, we excluded 356 nonprimary research articles (e.g., reviews, editorials, and case reports) and non-English publications, leaving 2,715 articles for full-text assessment. After excluding 29 articles for which the full text was unavailable, the remaining 2,686 articles were evaluated in detail. During this stage, 2,194 articles were excluded as they were not relevant to the core topic or lacked significant, extractable results concerning MSI in cancer. This screening process resulted in a final cohort of 492 research articles for data extraction.

#### Standardized data extraction and quality control

We used a multistage curation pipeline to standardize extracted data and perform cross-validation. First, all extracted data were standardized using established international guidelines: Gene symbols were harmonized according to the HUGO Gene Nomenclature Committee [19], and tumor types were mapped to the Disease Ontology [20] and the International Classification of Diseases, 11th Revision [21]. Second, a cross-validation process was performed by 2 independent researchers. Any discrepancies were resolved through consensus or consultation with a third senior reviewer. Third, each data entry was assigned a unique identifier for traceability and underwent a final validation check for completeness and accuracy before being integrated into the knowledgebase.

### Database architecture and implementation

#### Data schema design

Before data extraction, we defined a data framework to organize the main types of MSI-related evidence. Based on a preliminary review of representative literature and considerations of clinical relevance, we defined 4 primary feature categories (Table 1): (a) Genetic & Molecular Features (e.g., gene mutations, promoter methylation, and gene/protein expression); (b) Clinicopathological Features (e.g., tumor histology, grade, location, and patient demographics); (c) Therapeutic Response (e.g., response to immunotherapy, chemotherapy, and targeted therapy); and (d) Prognostic Factors (e.g., overall survival and disease-free survival).

**Table 1.** Summary of data categories and key fields in MSICKB

Data category	Description	Key data fields
Genetic & Molecular Features	Molecular alterations and genomic characteristics associated with MSI status	Gene symbol, Feature type (e.g., Mutation, Methylation, Expression), Molecular description (e.g., “frameshift mutation”)
Clinicopathological Features	Standardized patient demographic data and clinical/pathological characteristics of the tumors	Feature name, Feature type (e.g., Tumor location, Histology, Grade, Stage, Patient age, and Gender), Feature description
Therapeutic Response	Data detailing the outcomes of various treatments in the context of MSI status	Treatment type (e.g., Immunotherapy), Drug name (e.g., Pembrolizumab), End point (e.g., ORR and PFS), Response status (e.g., “better response” and “poor response”)
Prognostic Factors	Information linking specific features to patient survival outcomes	Prognostic factor name (e.g., “MSI-H” and “BRAF V600E”), Prognostic type (favorable/unfavorable), End point (e.g., OS and DFS)

MSICKB, Microsatellite Instability Cancer Knowledgebase; MSI, microsatellite instability; ORR, objective response rate; PFS, progression-free survival; OS, overall survival; DFS, disease-free survival

**Fig. 2.**
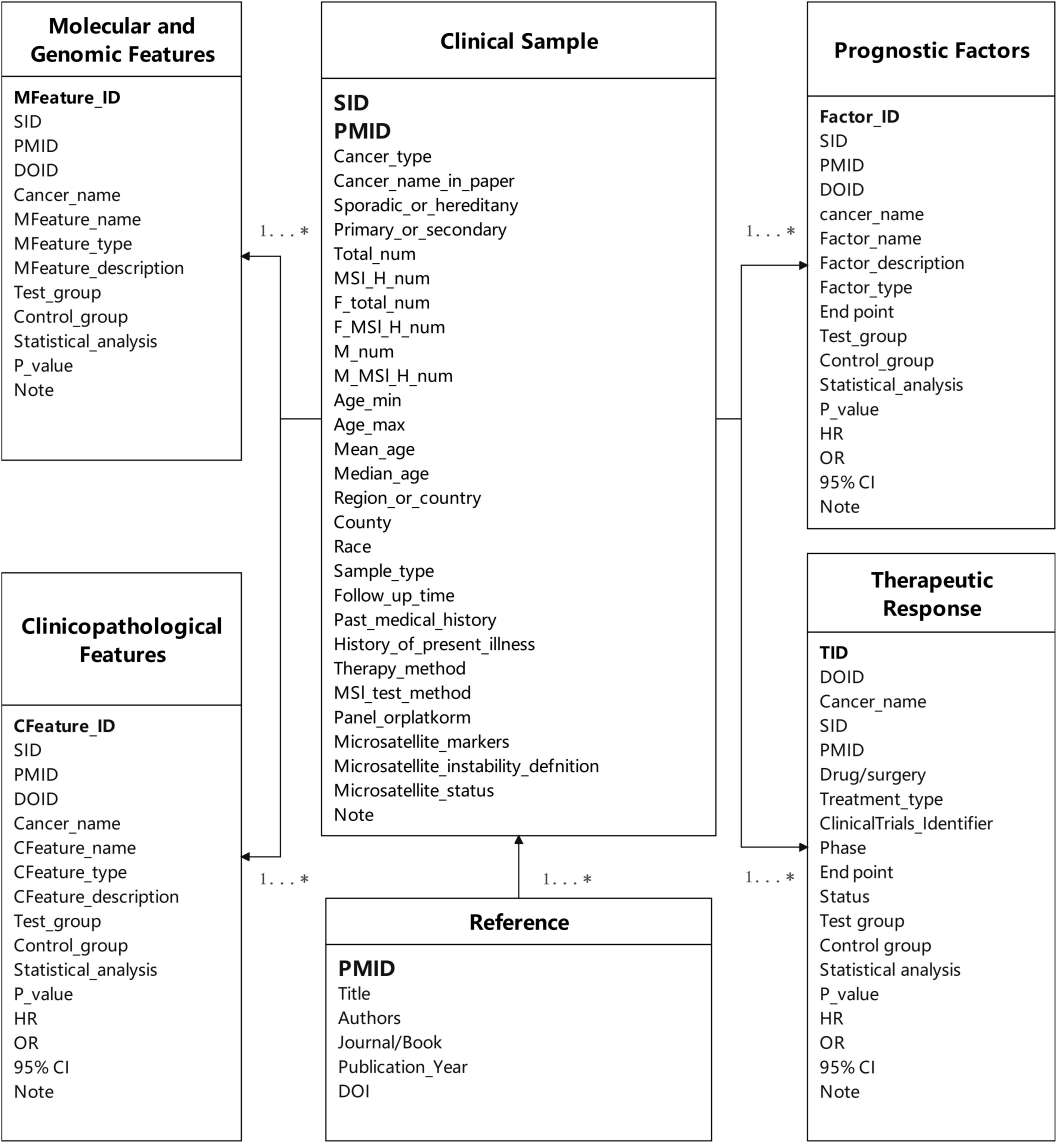
Entity–relationship (ER) diagram of the MSICKB database schema. The diagram shows the 6 core tables and their relationships. The Sample and Reference tables serve as central hubs, linked to the 4 feature-specific tables (Molecular and Genomic Features, Clinicopathological Features, Prognostic Factors, Therapeutic Response). The 1...* notation indicates a one-to-many relationship, where one record in a central table can be associated with multiple records in a feature table.

The knowledgebase was architected using a relational database model to ensure data integrity, minimize redundancy, and facilitate efficient querying. The core schema comprises 6 interconnected tables: a Reference table (bibliographic data), a Sample table (cohort details such as cancer type and sample size), and 4 feature-specific tables corresponding to the aforementioned categories (Fig. 2). Foreign keys link the feature tables to the Sample and Reference tables, enabling complex, multidimensional queries while maintaining data consistency.

#### Web implementation

The MSICKB was developed as a web-based platform using the XAMPP stack (v.8.2.12), which integrates Apache (v.2.4.58) as the web server, MySQL (v.8.2.12) as the relational database management system, and PHP (v.8.2.12) for server-side scripting. The front-end user interface was constructed with HTML5, CSS3, and JavaScript, incorporating the Bootstrap framework (v.4.6.2) to support responsive page layout across devices. For dynamic data visualization, we integrated the Pyecharts library (v.2.0.7), enabling interactive visualization of relationships within the curated dataset through the web interface. To guarantee high performance, especially for complex queries involving multitable joins, strategic indexing was applied to frequently accessed fields in the MySQL database. This architecture supports efficient querying and web-based exploration of the curated data.

### Network construction and analysis

A gene–cancer association network was constructed from the curated literature dataset by extracting unique gene–cancer pairs. For the primary analysis, we used a simple bipartite network in which each retained edge represented the presence of at least 1 curated publication supporting a given gene–cancer association. Degree-based network summaries and network visualization were generated in Python, including NetworkX (v3.2.1). The primary simple gene–cancer network was derived from 159 publications contributing at least 1 retained gene–cancer association.

Because MSI-H/microsatellite-stable status was not defined uniformly across studies, we recorded MSI testing information in MSICKB when available, including the reported test method (e.g., polymerase chain reaction [PCR], mismatch repair immunohistochemistry, and sequencing-based approaches), panel/platform, microsatellite markers, and MSI definition/positivity threshold [22]. To assess whether heterogeneity in MSI ascertainment could materially influence network-based degree summaries and hub identification, we performed an assay-stratified sensitivity analysis among the publications contributing to the primary simple gene–cancer network, as a robustness check rather than a full harmonization. Each publication was classified as PCR-only, immunohistochemistry-only, mixed/multiple, or unclear/not reported based on the original methods. Within each assay group, we rebuilt the simple gene–cancer network and reidentified hub genes using the same operational definition as in the primary analysis (degree ≥ 3). Robustness was summarized by the overlap and Jaccard index between the assay-specific top 9 hubs and the primary top-9 hub set.

To characterize the degree distribution of gene nodes, we fitted a power-law model using the Python package powerlaw with discrete=True, because gene degree is a discrete variable [23,24]. We also compared the power-law model with lognormal, exponential, and truncated power-law alternatives using likelihood ratio tests implemented in the same package. For the fitted tail, we report the estimated scaling exponent (*γ*), the lower bound of the fitted region (x_min), the Kolmogorov–Smirnov statistic, and an approximate 95% confidence interval (CI) for *γ*.

Highly connected genes were operationally defined as hub genes using a degree threshold of ≥3 in the primary simple network. This cutoff corresponded to the 90.9th percentile of the empirical degree distribution and was selected as a descriptive threshold to capture a sparse upper-tail subset while retaining a set large enough for downstream analyses. Sensitivity analyses were also performed using alternative thresholds of degree ≥2, 4, and 5. The association between hub status and cross-cancer universality was assessed using Fisher’s exact test, where universality was defined as presence in at least 2 cancer types. Odds ratios (ORs) and 95% CIs were reported; when a zero cell was present, Haldane correction was applied for effect-size estimation. As a supplementary sensitivity analysis, we also compared publication frequency between universal and nonuniversal genes using the Mann–Whitney U test and explored a penalized logistic regression model with universal status as the outcome and hub status together with log₂(PMID count + 1) as predictors; because hub status (degree ≥ 3) is definitionally nested within universal status (degree ≥ 2), this model was used only for sensitivity assessment rather than primary inference.

To assess the robustness of the network results to differences in supporting evidence, we performed sensitivity analyses based on the study-level evidence table used in the primary analysis, in which each row represented 1 unique gene–cancer association from 1 publication (gene–cancer–PubMed identifier [PMID]). Publication-level annotations for sample size and covariate adjustment were added. Adjustment was coded from the reported analytic methods as 0 for no adjustment, 1 for partial adjustment for 1 to 2 covariates, and 2 for multivariable analysis or adjustment for ≥3 covariates, and was used only as a rough proxy for confounding control rather than a formal study-quality score. For each sensitivity analysis, the evidence table was restricted according to the specified criterion, the corresponding subnetworks were rebuilt by collapsing retained rows to unique gene–cancer pairs, and the resulting top hub genes were compared with those from the primary simple network.

To further evaluate the influence of uneven literature coverage across cancer types, we calculated a cancer-weighted degree by assigning each gene–cancer edge a weight of 1/n_cancer, where n_cancer denotes the number of unique PMIDs available for that cancer in the study-level table, and summing these weights across cancers for each gene. We also performed a downsampling analysis in which cancer-specific publication counts above the upper quartile were capped at the upper-quartile value (10 in our data), PMIDs were randomly sampled to that cap, the network was rebuilt, and this procedure was repeated 1,000 times. The upper quartile was used as a simple data-driven threshold to define heavily studied cancers.

Functional enrichment analysis of the hub-gene set was performed using GSEApy through the Enrichr interface [25,26]. We queried the GO Biological Process, Kyoto Encyclopedia of Genes and Genomes, and WikiPathways gene-set libraries. The hub genes identified under the primary degree threshold (≥3) were used as the input gene set. All genes present in the primary simple network were specified as the background gene universe when supported by the queried library; however, because custom background submission was not supported in the final Enrichr runs, the reported enrichment statistics correspond to the default database background. For each term, we report the overlap, OR, raw *P* value, adjusted *P* value, combined score, and contributing genes. Benjamini–Hochberg false discovery rate (FDR) adjusted *P* values are provided in the full enrichment-results table, and adjusted *P* values were used to identify significantly enriched terms.

External molecular support for representative hub genes was evaluated using The Cancer Genome Atlas (TCGA) Pan-Cancer Atlas cohorts for endometrial (UCEC), colorectal (COADREAD), and gastric (STAD) cancers through the cBioPortal API [27–30]. Samples were stratified using the sample-level MSI_SENSOR_SCORE annotation, with MSI-H defined as a score of ≥3.5, and the remaining scored samples used as the comparison group. Mutation prevalence was compared using Fisher’s exact test, with Haldane correction applied for ORs and 95% CIs. Expression differences were assessed using the Mann–Whitney U test. For pooled analyses, mutation counts were combined across the 3 cohorts for a summary comparison, whereas expression *P* values were combined using Fisher’s method. Multiple testing was controlled using the Benjamini–Hochberg procedure.

## Results

### Web interface and data accessibility

**Fig. 3.**
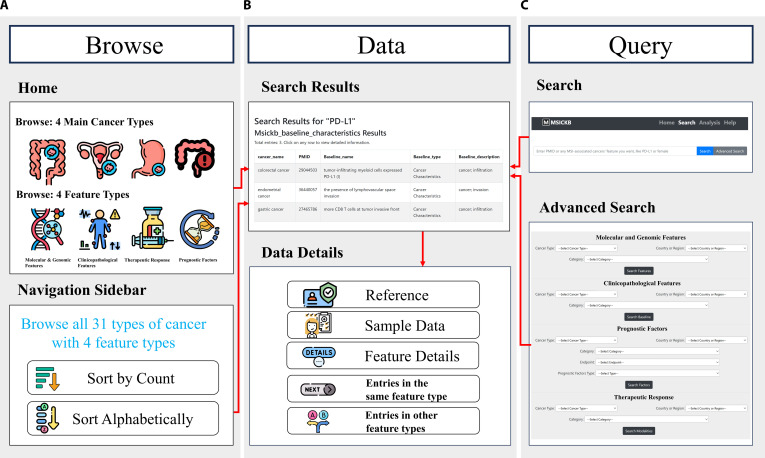
Workflow and functional architecture of the MSICKB web portal. The platform follows a “Browse/Query-to-Data” logic flow. (A) Browse Mode: Users can navigate via visualized icons or the Navigation Sidebar, which supports hierarchical expansion of cancer types and feature categories. (B) Data Presentation: The data view integrates search results with a comprehensive Details Page. The logic flow (red arrows) demonstrates how specific entries link to reference metadata and cross-referenced features from the same publication, facilitating context-aware data exploration. (C) Query Mode: The search interface supports quick lookups and Advanced Search for filtering by cancer type, category, region, etc.

The MSICKB is freely accessible at http://www.sysbio.org.cn/MSICKB/. MSICKB provides a web portal for both browsing and targeted queries (Fig. 3).

For broad exploration, the Browse module (Fig. 3A) serves as the main browsing interface. In addition to graphical entry points for major cancer types and 4 functional dimensions, a hierarchical Navigation Sidebar provides a structured tree view, allowing users to systematically expand specific cancer types to access their corresponding feature subcategories directly.

For targeted queries, the Query module (Fig. 3C) provides both quick keyword search and an Advanced Search interface. Users can construct multidimensional queries by strictly filtering for cancer types, feature subcategories, geographical regions, and domain-specific parameters (e.g., clinical end points). This ensures the precise retrieval of specific molecular or clinical features derived from curated literature, rather than broad document matching.

Finally, results from both browse and query functions are displayed through a common data presentation interface (Fig. 3B). The search results display essential metrics (e.g., *P* value, hazard ratio, and 95% CI), and links to detailed supporting records. The details page provides the associated Reference (linked via PMID) and Sample information for each entry. The system also displays additional molecular or clinical features reported in the same publication. This helps users review multiple MSI-related features reported within the same study cohort. Furthermore, a dedicated Help page provides detailed documentation to ensure ease of use.

### Data content and hierarchical structure

**Fig. 4.**
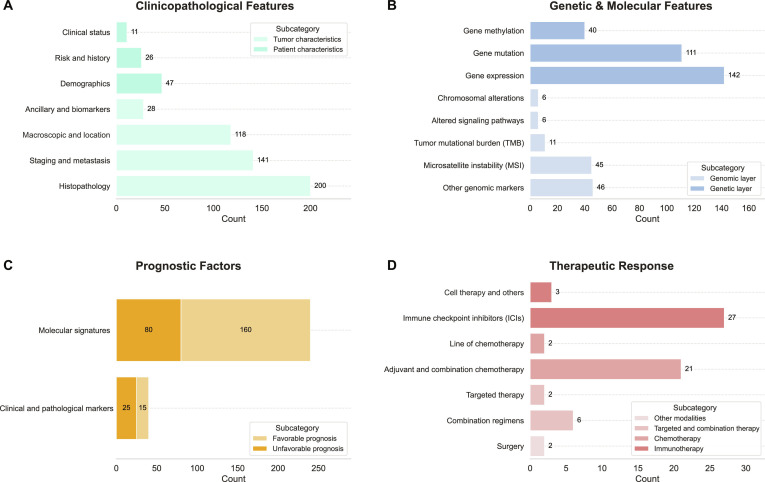
Hierarchical composition of the 4 primary feature domains in MSICKB. The bar charts display the frequency of features within the subcategories of (A) Clinicopathological Features, (B) Genetic & Molecular Features, (C) Prognostic Factors, and (D) Therapeutic Response. Colors within each panel correspond to second-level subcategories with counts annotated for each bar.

As of its current release, MSICKB consolidates a total of 1,382 standardized features meticulously curated from 492 peer-reviewed publications, spanning 31 cancer types. These features are organized according to a 4-level hierarchical annotation framework, visualized in the tree map (Fig. 4). At the top level, the data are divided into 4 primary domains: Clinicopathological Features (*n* = 606, 44%), Genetic & Molecular Features (*n* = 412, 26%), Prognostic Factors (*n* = 301, 20%), and Therapeutic Response (*n* = 63, 5%). This structure converts heterogeneous literature evidence into a standardized, queryable format, enabling users to perform targeted queries at different granularities. Further analyses, detailed in the Supplementary Materials, reveal a heterogeneous distribution of these features across cancer types (Fig. S1) and distinct spatiotemporal trends in the research landscape (Fig. S2).

### Network analysis reveals a heavy-tailed and functionally coherent core

**Fig. 5.**
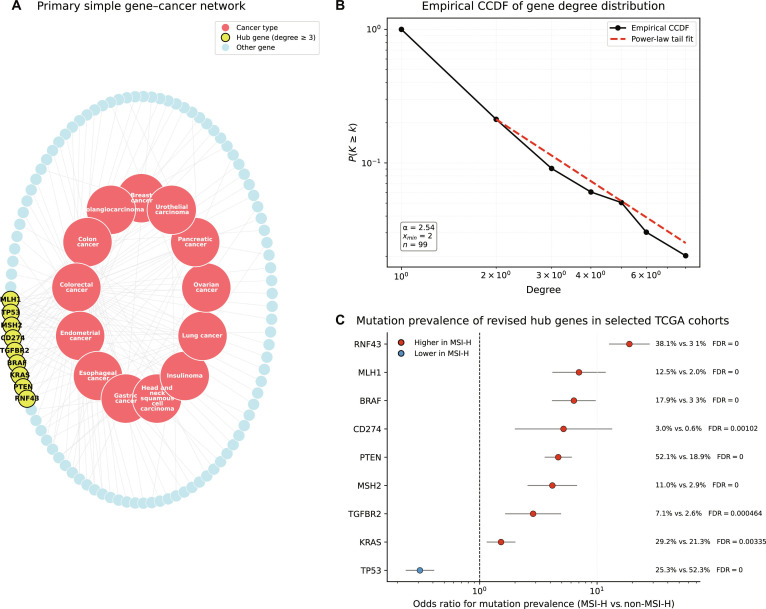
Gene–cancer network architecture and external support for hub genes. (A) Primary simple gene–cancer network constructed from curated MSICKB associations. Each edge represents a retained gene–cancer association in the primary simple network. Hub genes were operationally defined as genes connected to at least 3 cancer types (degree ≥ 3) and are highlighted. (B) Empirical complementary cumulative degree distribution (CCDF) of gene degree in the primary simple network, shown together with the fitted power-law tail. (C) Pooled mutation support for hub genes across selected TCGA MSI-associated cohorts (endometrial, colorectal, and gastric cancers). Points indicate odds ratios for mutation prevalence in MSI-H versus non-MSI-H samples, with horizontal lines indicating 95% confidence intervals. Multiple testing was controlled using the Benjamini–Hochberg procedure.

The primary simple gene–cancer network comprised 99 genes, 13 cancer types, and 147 unique edges. Gene degree, defined as the number of distinct cancer types connected to each gene, was highly right-skewed (median = 1, mean = 1.48, maximum = 8; Fig. S4), indicating that most genes were linked to only a single cancer type, whereas a small subset showed broader cross-cancer connectivity. Fitting the degree distribution yielded an estimated exponent of γ = 2.54 (95% CI: 1.88 to 3.20), with x_min = 2 and KS = 0.045 (Fig. 5B). However, likelihood-ratio comparisons did not show a statistically significant preference for the power-law model over lognormal, exponential, or truncated power-law alternatives (Table S6). We therefore describe the network as having a heavy-tailed degree distribution rather than a strict scale-free topology.

Using an operational cutoff of degree ≥ 3 (90.9th percentile), we identified 9 hub genes: BRAF, CD274, KRAS, MLH1, MSH2, PTEN, RNF43, TGFBR2, and TP53 (Fig. 5A). Sensitivity analyses using stricter thresholds (degree ≥ 4 and ≥ 5) retained a stable core subset of highly connected genes (Table S7), indicating that the primary hub signal was robust to reasonable cutoff choices. All 9 hub genes also met the broader universality criterion (degree ≥ 2), whereas only 12 of the remaining 90 nonhub genes did so (Fisher’s exact test, *P* = 1.70 × 10^−7^; Haldane-corrected OR = 119.3, 95% CI: 6.53 to 2,181). However, universal genes also had higher supporting publication counts than nonuniversal genes (Mann–Whitney U, *P* = 1.49 × 10^−16^; Fig. S7), indicating that this pattern should be interpreted cautiously. A publication-adjusted penalized logistic sensitivity model is provided in Table S10, although its coefficients should be interpreted cautiously because hub status is definitionally nested within universal status.

Additional sensitivity analyses supported the robustness of the hub structure. Across the publications contributing to the primary simple network, PCR-only studies constituted the majority of the available MSI testing evidence (Fig. S3). Assay-stratified hub sets showed partial overlap with the primary top-9 hub set, and a narrower sensitivity analysis restricted to studies with broadly similar PCR-based MSI definitions retained 6 of the 9 primary hub genes (Tables S1 and S2). Restricting the included studies to larger studies (sample size ≥ median = 145) retained 8 of the 9 primary hub genes (Jaccard = 0.80), whereas a stricter restriction (sample size ≥ Q3 = 593) retained 6 of 9 hubs (Jaccard = 0.50). Restricting the analysis to studies reporting at least some covariate adjustment retained 8 of 9 hubs for adjustment ≥ 1 and 7 of 9 hubs for adjustment = 2 (Table S3). To examine whether uneven literature volume across cancers could influence degree-based summaries, we performed 2 additional sensitivity analyses. Using degree weighted by cancer-specific publication counts, the top-9 set overlapped 6 of 9 genes with the standard degree-based top-9 list (Jaccard = 0.50; Table S4). After downsampling the most heavily studied cancers across 1,000 iterations, the resulting networks retained a mean overlap of 7.56 of 9 hubs with the primary top-9 set (median 8 of 9; mean Jaccard = 0.730, median = 0.800; Table S5).

Functional enrichment analysis of the hub-gene set showed convergence on MSI-relevant and cancer-related pathways, particularly DNA mismatch repair/genomic instability and cancer-associated pathways (Fig. S5 and Table S8). Gene degree was strongly correlated with publication frequency across the network (Spearman ρ = 0.84, *P* < 0.001; Fig. S6), indicating that the enrichment results should be interpreted with caution in a literature-derived resource. Additional descriptive comparisons using simple gene-level publication-based rankings showed partial overlap with the primary hub set but did not alter the primary network definition (Table S9). These results are interpreted as descriptive functional summaries of the hub-gene set. To provide external molecular support for the knowledgebase-derived hub genes, we further compared somatic mutation prevalence and mRNA expression between MSI-H and non-MSI-H tumors across 3 TCGA cohorts (endometrial, colorectal, and gastric cancers; total n = 1,550). In per-cohort analyses, 5 to 8 of the 9 hub genes showed significantly differential mutation prevalence and/or expression after FDR correction, depending on cancer type (Tables S11 and S13). In pooled analyses (n = 336 MSI-H and n = 1,214 non-MSI-H tumors), all 9 hub genes showed significantly differential mutation prevalence and expression (FDR < 0.05 for all). Eight genes were more frequently mutated in MSI-H tumors, whereas TP53 showed an inverse association (Fig. 5C). Representative pooled effect sizes (Table S12) were large for RNF43 (OR = 18.83, 95% CI: 12.76 to 27.79) and MLH1 (OR = 7.01, 95% CI: 4.20 to 11.72), whereas TP53 showed lower mutation prevalence in MSI-H tumors (OR = 0.31, 95% CI: 0.24 to 0.41). At the expression level, reduced MLH1 expression and elevated CD274 expression in MSI-H tumors were among the most prominent pooled patterns (Table S14). Overall, these analyses support MSICKB as a resource for structured integration, systematic validation, and prioritization of established MSI-associated genes, rather than as a platform for claiming novel mechanistic discovery.

### Comparison with existing databases

To position MSICKB within the current data landscape, we compared its features with major cancer genomics repositories such as TCGA, cBioPortal, and COSMIC (Catalogue of Somatic Mutations in Cancer) [15,27,30] (Table 2). The comparison highlights the distinct role of MSICKB in providing manually curated, literature-traceable, feature-level clinical outcome data with a specific focus on the MSI context.

**Table 2.** Comparison of MSICKB with existing major cancer databases

Feature	MSICKB (this work)	TCGA/GDC Portal	cBioPortal	COSMIC
Primary Data Source	Manually curated from peer-reviewed literature	Raw multiomics data (e.g., WES, RNA-seq)	Raw multiomics data (TCGA, ICGC, etc.)	Literature curation and sequencing data
Focus	Disease-specific: Microsatellite instability (MSI) cancers	Pan-cancer genomic and transcriptomic profiles	Pan-cancer genomic data visualization	Pan-cancer somatic mutations
Data Granularity	Evidence-based, feature-level knowledge (e.g., “Gene X predicts poor prognosis in MSI-H CRC”)	Gene/protein expression levels, mutation calls, copy number variations	Gene-level alterations and clinical data	Mutation-level annotations
Core Content Dimensions	Integrates 4 key dimensions:	Primarily genomic, transcriptomic, and basic clinical data	Primarily genomic and basic clinical data	Primarily somatic mutations and basic clinical data
	1. Genetic & Molecular			
	2. Clinicopathological			
	3. Therapeutic Response			
	4. Prognostic Factors			
Therapeutic Data	Rich, curated details on drug response, especially immune checkpoint inhibitors (ICIs)	Limited and not standardized across studies	Limited; mainly focused on sample metadata	Limited drug resistance mutation data
Prognostic Data	Explicitly curated prognostic associations (e.g., HR, OS, and DFS)	Requires reanalysis of survival data	Basic survival plots available	Limited prognostic information
User Accessibility	Intuitive web interface with direct search/browse functions	Data portal for file download and exploration	User-friendly visualization and analysis tools	Searchable web interface
Integrated Analysis Tools	Built-in network analysis application example	Basic visualization tools (e.g., OncoGrid)	OncoPrint, survival analysis, coexpression	None

MSICKB, Microsatellite Instability Cancer Knowledgebase; MSI-H, MSI-high; TCGA, The Cancer Genome Atlas; GDC Portal, NCI Genomic Data Commons Data Portal; COSMIC, Catalogue of Somatic Mutations in Cancer; WES, whole-exome sequencing; RNA-seq, RNA sequencing; ICGC, International Cancer Genome Consortium; HR, hazard ratio; OS, overall survival; DFS, disease-free survival

## Discussion

MSICKB provides a curated, literature-based resource for organizing MSI-associated molecular evidence across cancers and enabling cross-cancer comparison of reported gene–cancer associations. In this context, the network analysis is most appropriately interpreted as a structured integration and prioritization of MSI-associated signals captured by the literature, rather than as a platform for claiming novel mechanistic discovery. The identified hub genes and their associated pathways are largely consistent with established MSI biology, supporting the value of the resource as a systematic synthesis of the field [6–8,12,13].

Several limitations should be considered when interpreting the network analyses. First, the current network is moderate in size, which limits statistical power for topology inference. Accordingly, the observed heavy-tailed degree distribution should not be interpreted as definitive evidence of a strict scale-free architecture, but rather as indicating a nonuniform degree pattern with a small subset of broader cross-cancer hubs and remaining compatible with multiple plausible tail distributions [24,31]. Second, hub definitions depend on an operational cutoff; although the main hub pattern remained broadly stable across reasonable alternative thresholds, no single threshold should be considered uniquely correct. Third, because the primary network is a simple graph with 1 edge per unique gene–cancer pair, it summarizes cross-cancer association breadth rather than the strength or quality of supporting evidence. Sensitivity analyses restricted by MSI assay type, study size, and covariate adjustment supported the robustness of the main hub pattern, but these checks are supportive rather than definitive and do not replace a formal weighted evidence synthesis. Finally, because MSI status was not defined uniformly across all studies, some heterogeneity in MSI ascertainment is unavoidable in a literature-derived resource, and this should be kept in mind when interpreting cancer-specific associations, especially in precision oncology applications.

An additional limitation is that MSICKB is derived from MSI-focused published literature and therefore partly reflects research intensity. Genes that are more frequently studied have more opportunities to be reported across multiple cancer types, potentially inflating network connectivity. Consistent with this concern, gene degree was strongly correlated with publication frequency (Spearman ρ = 0.84, *P* < 0.001), and universal genes also had higher publication counts than nonuniversal genes. Uneven literature volume across cancer types may likewise influence degree-based summaries. Sensitivity analyses using cancer-weighted degree and downsampling of heavily studied cancers showed that the main hub pattern was broadly retained, although hub rankings were not identical. We therefore do not interpret the heavy-tailed degree pattern as evidence of an intrinsic hierarchical organization. Bias-aware null models were not implemented in this revision and remain a direction for future methodological work. For these reasons, enrichment of hub genes in canonical MSI-related pathways should be interpreted as a descriptive summary of the MSI literature captured by MSICKB, rather than as independent validation. Similarly, cross-cancer universality should not be interpreted as a purely biological property, because it also reflects the current distribution of attention within the literature. As the field expands to include less-studied malignancies, the apparent universality landscape may also evolve.

Despite these limitations, the TCGA-based analyses provided orthogonal molecular support for the literature-derived hub set, with all 9 hub genes showing significant pooled differences in mutation prevalence and expression between MSI-H and non-MSI-H tumors across 3 MSI-associated cancer types [12,13,29,30]. These findings complement the curated literature network while not removing the interpretive constraints inherent to a literature-derived resource. As MSICKB expands, future work may enable more definitive topological characterization, more explicit bias-aware network modeling, extension to clinically oriented end points such as prognosis, treatment response, and immunotherapy-related stratification [4,9,32], and broader integration with multimodal molecular and clinical data. More broadly, structured integration of MSI-associated evidence may support biomarker-guided research prioritization and translational use of MSI-related knowledge in precision oncology, particularly as MSI testing and biomarker-guided treatment strategies continue to expand across cancer types.

## Conclusion

In conclusion, MSICKB provides a curated framework for organizing and comparing MSI-associated molecular evidence across cancers. The resulting network analyses highlight a nonuniform cross-cancer landscape with a limited set of broadly connected hub genes, supported in part by orthogonal TCGA-based molecular analyses. While the findings should be interpreted cautiously in light of the literature-derived design and related methodological constraints, MSICKB offers a useful starting point for MSI-related evidence synthesis, biomarker prioritization, and future integrative studies.

## Data Availability

The code and study materials are available at https://github.com/ExplorerZhyuxin/MSICKB. A time-stamped, versioned snapshot of the MSICKB data used in this study is archived on Zenodo (DOI: https://doi.org/10.5281/zenodo.19042834). The MSICKB website also provides interactive query and bulk download. Considering the pace of MSI-related literature growth and the manual effort required for curation, we plan to update the database approximately every 2 years. Each release is versioned and accompanied by release notes. Programmatic access (API) is not currently provided and will be considered in future releases.
