# Supplementary material for: MSICKB: A Curated Knowledgebase for Exploring Molecular Heterogeneity and Biomarker Prioritization in Microsatellite Instability Cancers
Source: Comput Struct Biotechnol J. 2026 Apr 20;35(1):0047. doi: 10.34133/csbj.0047 (PMC13094419; doi:10.34133/csbj.0047)
Supplement: Supplementary 1 — Figs. S1 to S7 Tables S1 to S14 [file csbj.0047.f1.zip › Supplementary Material.pdf]

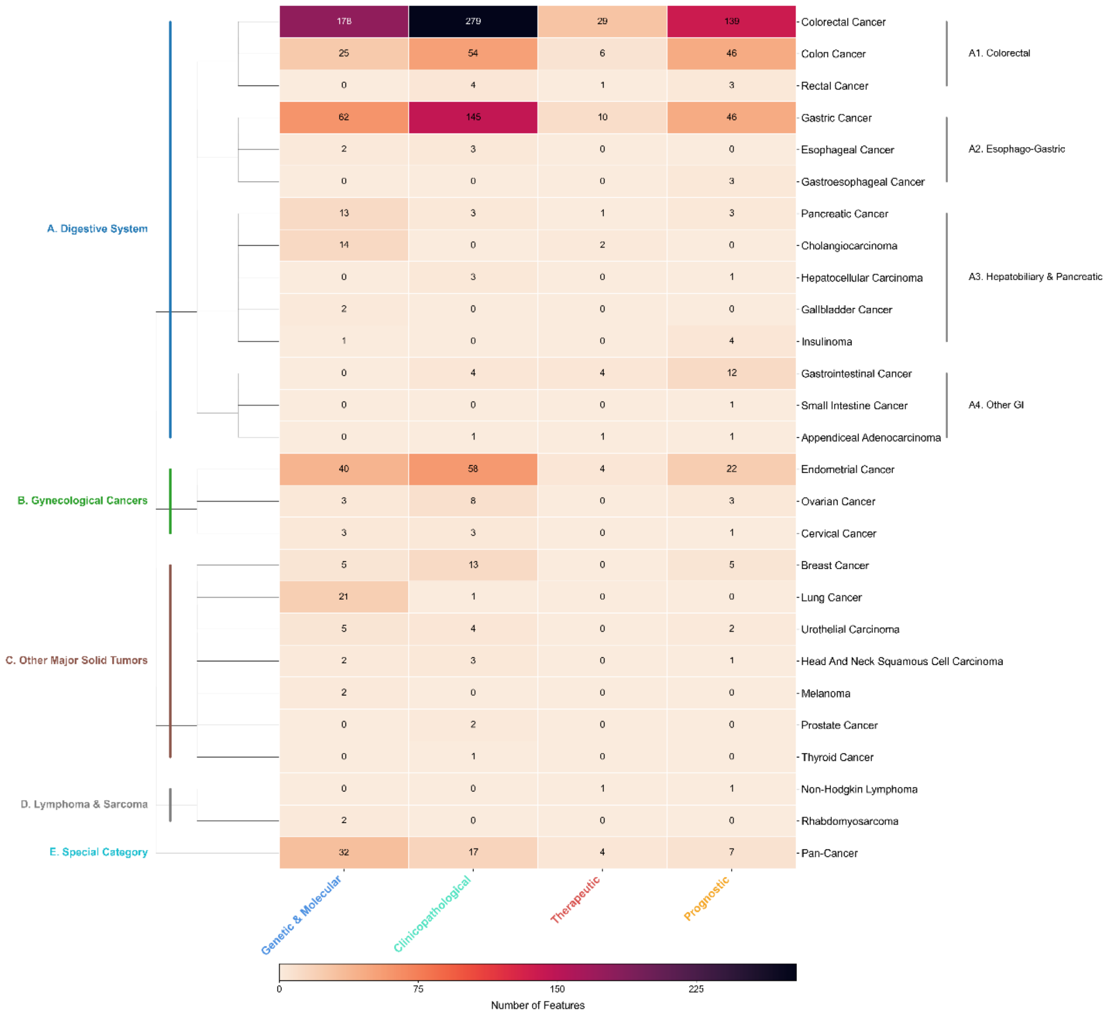

	Genetic & Molecular	Clinicopathological	Therapeutic	Prognostic
Colorectal Cancer	178	279	29	139
Colon Cancer	25	54	6	46
Rectal Cancer	0	4	1	3
Gastric Cancer	62	145	10	46
Esophageal Cancer	2	3	0	0
Gastroesophageal Cancer	0	0	0	3
Pancreatic Cancer	13	3	1	3
Cholangiocarcinoma	14	0	2	0
Hepatocellular Carcinoma	0	3	0	1
Gallbladder Cancer	2	0	0	0
Insulinoma	1	0	0	4
Gastrointestinal Cancer	0	4	4	12
Small Intestine Cancer	0	0	0	1
Appendiceal Adenocarcinoma	0	1	1	1
Endometrial Cancer	40	58	4	22
Ovarian Cancer	3	8	0	3
Cervical Cancer	3	3	0	1
Breast Cancer	5	13	0	5
Lung Cancer	21	1	0	0
Urothelial Carcinoma	5	4	0	2
Head And Neck Squamous Cell Carcinoma	2	3	0	1
Melanoma	2	0	0	0
Prostate Cancer	0	2	0	0
Thyroid Cancer	0	1	0	0
Non-Hodgkin Lymphoma	0	0	1	1
Rhabdomyosarcoma	2	0	0	0
Pan-Cancer	32	17	4	7

Supplementary Figure S1. Heatmap of feature distribution across cancer types. *The heatmap displays the number of features in each of the four main categories across 31 cancer types. Cancer types (y-axis) are organized according to a predefined, organ-system-based classification scheme, revealing five major super-clusters (labeled A-E on the left). The analysis reveals a strikingly heterogeneous landscape of MSI research. A significant disparity exists between the pervasive reporting of mechanistic-level features ('Genetic & Molecular' and 'Clinicopathological') across most cancers and the concentration of clinical-outcome features ('Prognostic' and 'Therapeutic') in a few well-characterized malignancies, most notably Colorectal Cancer. This imbalance highlights a critical translational gap in the current research landscape.*

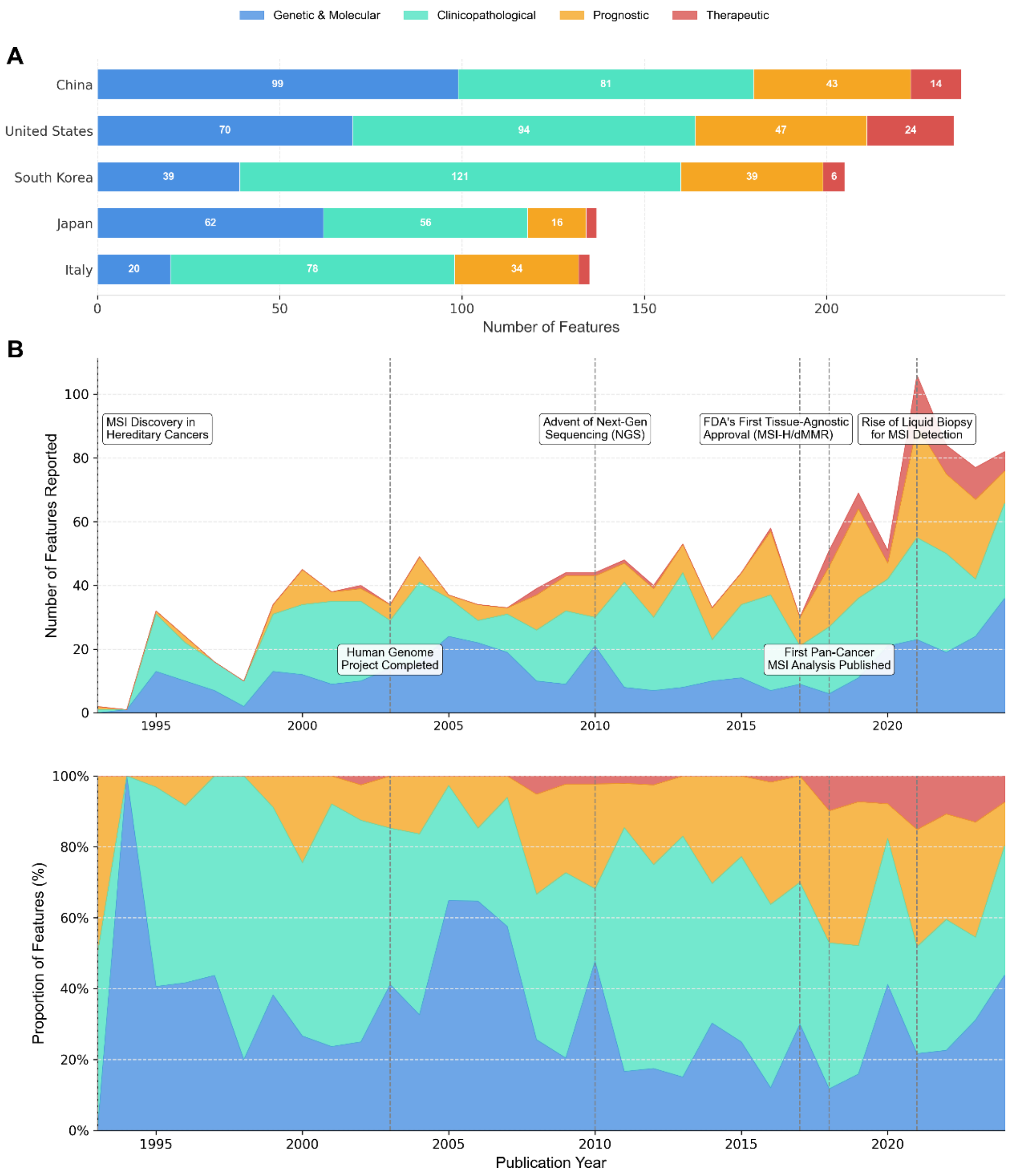

Supplementary Figure S2. Spatiotemporal evolution of MSI research.

(A) Geographical distribution of MSI-related features from top contributing countries. *The stacked bar chart illustrates the total number of curated features from the top eight countries and the proportional distribution of the four primary feature categories within each country. The analysis highlights distinct national research focuses, such as the prevalence of "Genetic & Molecular" studies from China versus the focus on "Clinicopathological" data from the United States and South Korea.* ***(B) Temporal trend of MSI-related research from 1995 to 2024.*** *The upper panel shows the absolute number of new features reported annually, annotated with key historical milestones (e.g., NGS advent, pan-cancer analysis). The lower panel displays the proportional distribution of the four feature categories over time, demonstrating a clear paradigm shift from early mechanistic exploration towards recent clinical translation. This trend, however, is not uniform across all cancer types, reflecting the translational gap highlighted in Supplementary*

Figure S1.

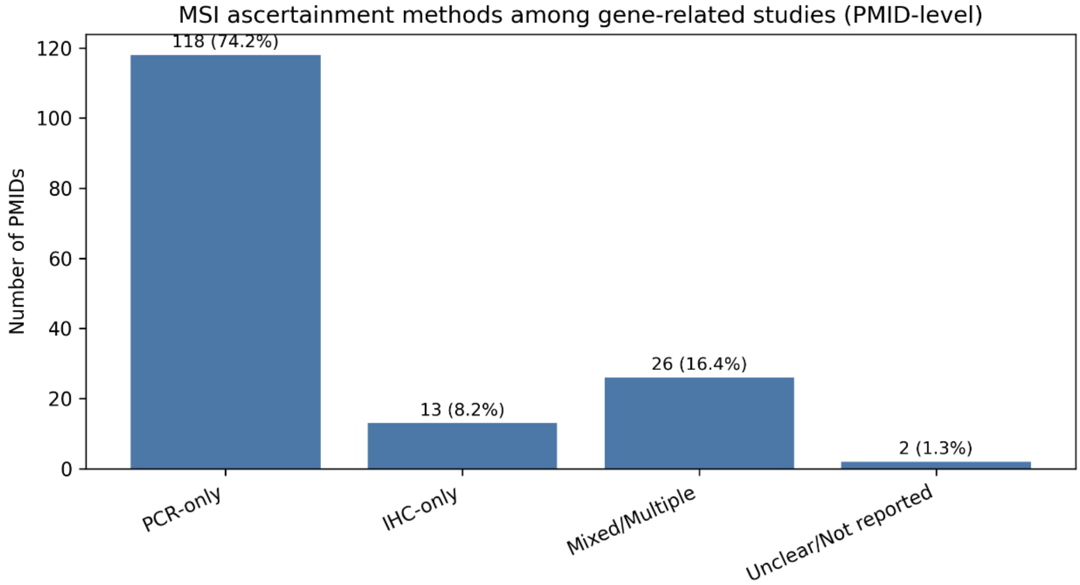

Supplementary Figure S3. MSI testing methods in the studies used for the gene–cancer network.

Bar plot showing how MSI status was assessed in the 159 publications contributing to the primary simple gene–cancer network. Each publication was classified as PCR-only, IHC-only, mixed/multiple, or unclear/not reported based on the original study methods. Most studies used PCR only.

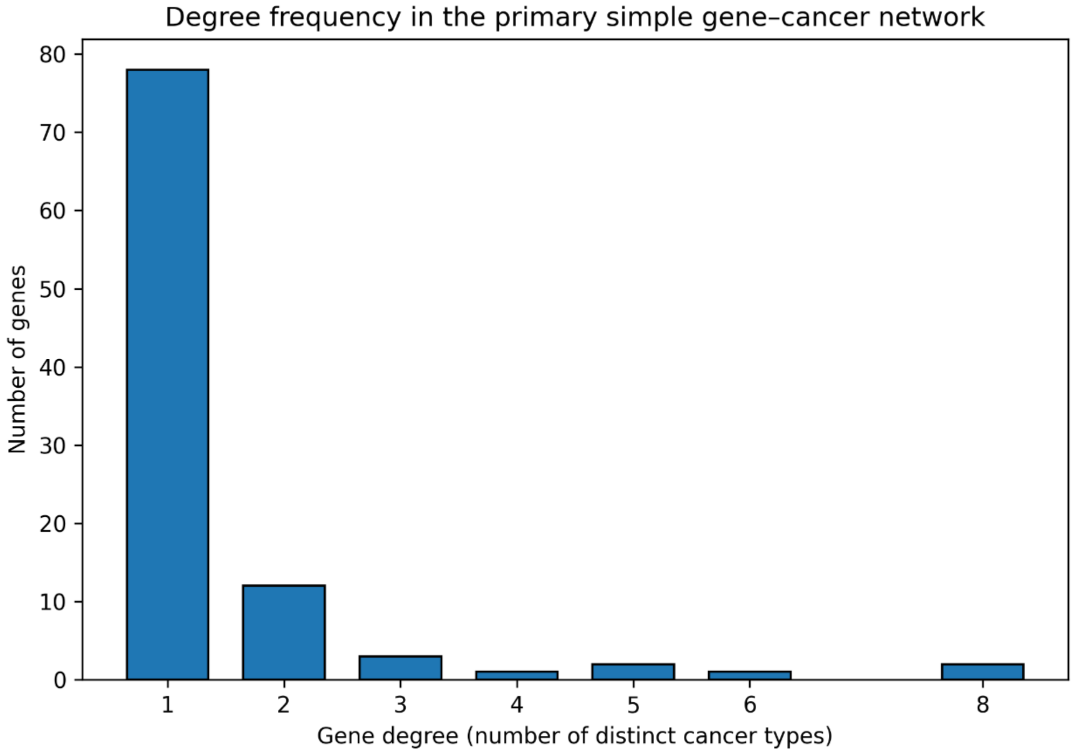

Supplementary Figure S4. Degree frequency distribution of genes in the primary simple gene–cancer network

Bar plot showing the frequency of gene degree values in the primary simple gene–cancer network. Degree was defined as the number of distinct cancer types connected to each gene. The distribution is strongly right-skewed, with most genes linked to a single cancer type and a smaller subset showing broader cross-cancer connectivity.

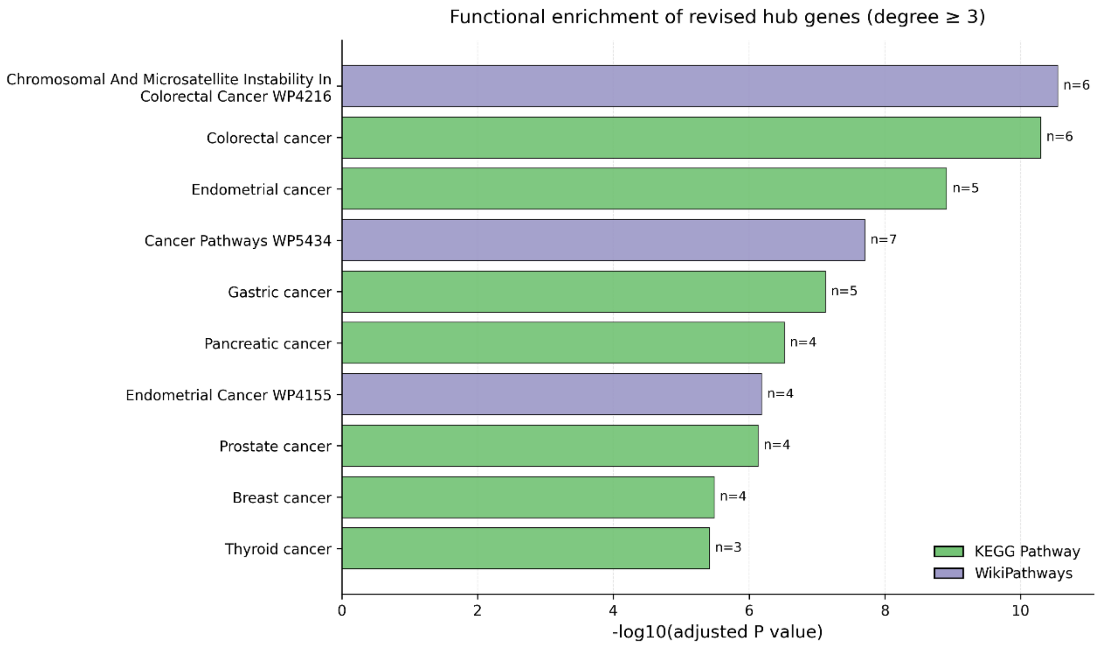

Supplementary Figure S5. Functional enrichment of revised hub genes (degree ≥ 3)

Bar plot showing the top enriched pathways for the nine revised hub genes (degree ≥ 3) based on over-representation analysis against KEGG and WikiPathways databases. The x-axis represents $-\log_{10}$*(adjusted p-value). Pathway sources are color-coded: green for KEGG, purple for WikiPathways. The number of overlapping hub genes is indicated for each term.*

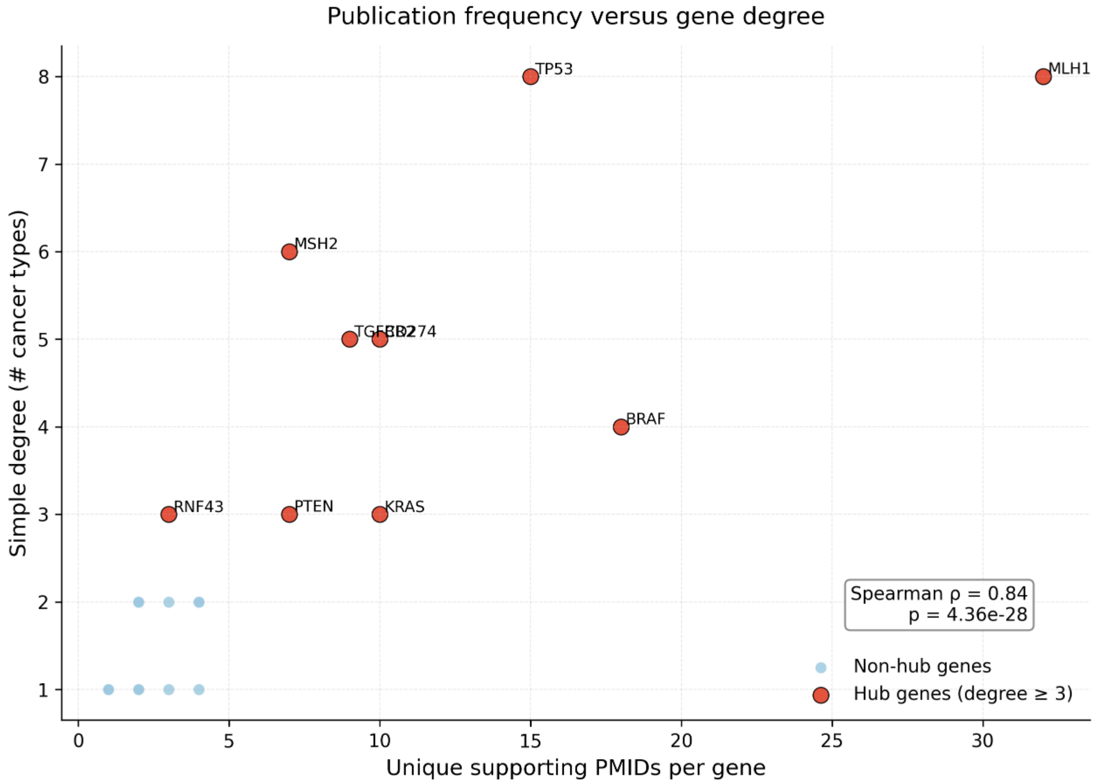

Supplementary Figure S6. Relationship between gene degree and publication frequency

Scatter plot showing the relationship between simple gene degree and the number of unique supporting PMIDs per gene in the revised gene–cancer network. Hub genes are highlighted for visualization. The strong positive correlation (Spearman ρ = 0.84, p < 0.001) indicates that highly connected genes also tend to be more frequently studied in the MSI literature.

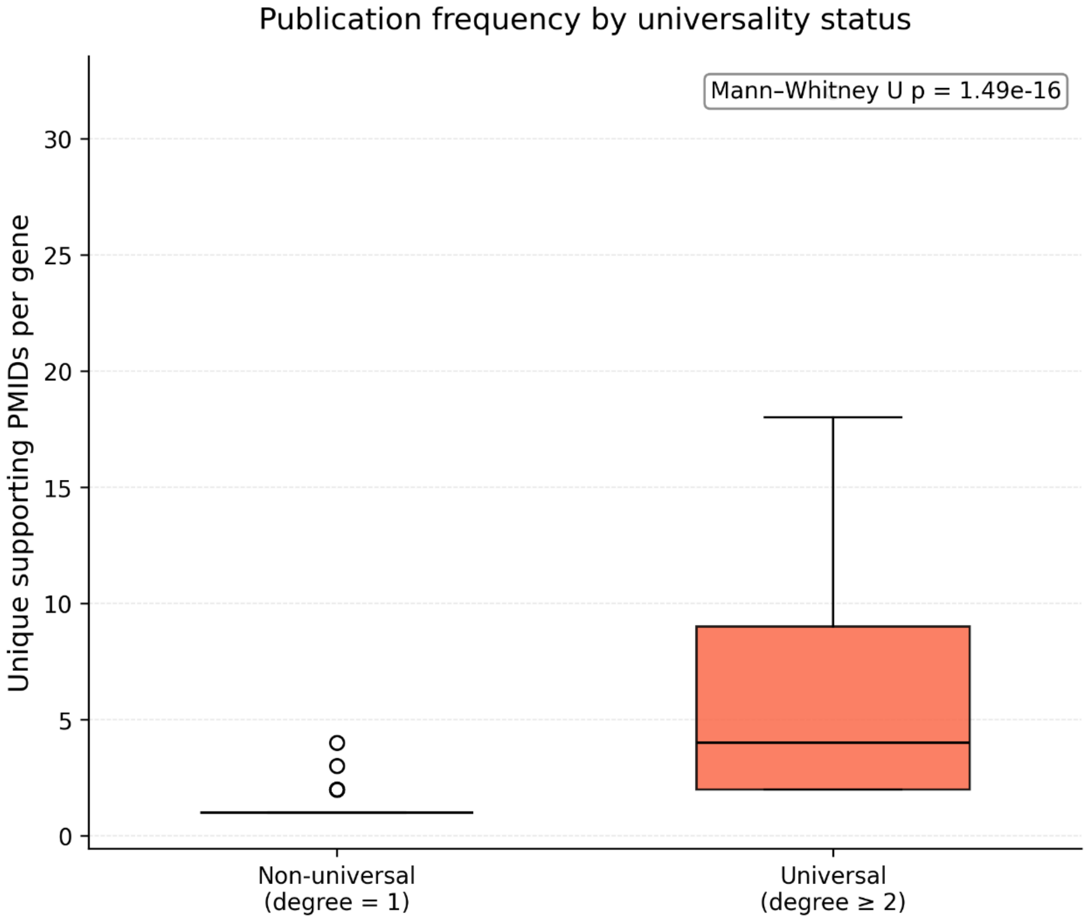

Supplementary Figure S7. Publication frequency by gene universality status

Boxplot comparing the number of unique supporting PMIDs between universal genes (degree ≥ 2, associated with multiple cancer types) and non-universal genes (degree = 1). Universal genes had significantly higher publication frequencies (Mann–Whitney U, $p = 1.49 \times 10^{-16}$), indicating that cross-cancer associations are partly driven by research intensity.

Supplementary Table S1. Hub-gene overlap in assay-specific subsets

Assay group	n publications	n unique gene–cancer edges	Top 9 hubs in subset	Overlap with main top-9 hubs	Jaccard
PCR-only	118	114	MLH1, MSH2, TP53, BRAF, KRAS, TGFBR2, CTNNB1, CD274, PIK3CA	7/9	0.636
Mixed/multiple	26	37	MLH1, TP53, MSH2, PTEN, ATG7, CCNA2, CD274, COX-2, CDH1	5/9	0.385
IHC-only	13	28	CD274, BRAF, ACVR2A, MLH1, B2M, DAPK1, ARID1A, FAM167A, HOXC6	3/9	0.200

Comparison of the top 9 hub genes from PCR-only, mixed/multiple, and IHC-only subsets with the main top-9 hub set from the primary simple gene–cancer network. Overlap is shown as the number of shared genes and the Jaccard index.

Supplementary Table S2. Sensitivity analysis restricted to studies using a common PCR MSI definition

Subset	n publications	n unique gene–cancer edges	Top 9 hubs	Overlap with main top-9 hubs	Jaccard
Studies matched to sample-level records with PCR-related MSI definitions reported as "≥2 of 5 unstable markers" or similar wording	47	52	MLH1, PIK3CA, BRAF, COX-2, TP53, TGFBR2, PTEN, TYMS, KRAS	6/9	0.50

Summary of an additional PCR-related sensitivity analysis using studies matched to sample-level records with similar reported MSI-definition wording. Additional sheets in the workbook list the included publications and the matched sample-level definition records.

Supplementary Table S3. Sensitivity analyses of hub identification under evidence subsets defined by study size and statistical adjustment

Subset	n publications	n study-level rows	n unique gene–cancer edges	n genes	n cancers	Top 9 hubs	Overlap with main top-9 hubs	Jaccard
Reference (primary simple network)	147	231	147	99	13	MLH1, TP53, MSH2, TGFBR2, CD274, BRAF, PTEN, RNF43, KRAS	9/9	1.00
sample size ≥ median (145)	67	115	86	60	8	CD274, TGFBR2, TP53, BRAF, PTEN, MLH1, RNF43, KRAS, BRCA2	8/9	0.80
sample size ≥ Q3 (593)	22	58	55	40	6	TGFBR2, CD274, RNF43, BRAF, TP53, MSH2, ARID1A, BRCA2, KMT2D	6/9	0.50
adjustment ≥ 1	66	102	74	60	7	MLH1, CD274, BRAF, TGFBR2, PTEN, TP53, MSH2,	8/9	0.80

adjustment = 2	56	87	65	54	7	KRAS, ATM MLH1, CD274, TP53, PTEN, MSH2, BRAF, TGFBR2, B2M, CDX2	7/9	0.636

Subnetworks were built by restricting the study-level evidence table (unique gene–cancer–PMID) to the specified subset of publications and then collapsing the retained rows to unique gene–cancer edges. If more than one quality record was available for the same PMID, the maximum value was used for sample size and adjustment. Hub genes were defined as the top 9 genes by degree in each subnetwork.

Supplementary Table S4. Comparison of standard degree and degree weighted by cancer-specific publication counts

Gene	degree	weighted degree
MLH1	8	4.098
TP53	8	1.948
RNF43	3	1.750
TGFBR2	5	1.651
ARID1A	2	1.500
MSH2	6	1.348
CD274	5	1.348
KMT2D	2	1.250
ACVR2A	2	1.037

Top 9 genes ranked by degree weighted by cancer-specific publication counts. Weighted degree was defined as the sum, across connected cancers, of 1 / n_cancer, where n_cancer is the number of unique PMIDs contributing to that cancer in the study-level primary-analysis table.

Supplementary Table S5. Downsampling analysis for highly studied cancers

Metric	Value
Iterations	1,000
Downsampling rule	Cancers with n publications > Q3 were capped at Q3
Q3 cap	10 publications
High-volume cancers	Colorectal Cancer; Gastric Cancer; Endometrial Cancer
Mean overlap with main top-9 hubs	7.56/9
Median overlap with main top-9 hubs	8/9
Mean Jaccard	0.730
Median Jaccard	0.800
Jaccard range	0.500–1.000

Summary of 1,000 random downsampling iterations in which cancers with publication counts above the upper quartile were capped at the upper-quartile value, while other cancers were left unchanged. Additional sheets report the per-cancer downsampling plan, main-hub retention frequencies, all hub frequencies, and iteration-level results.

Supplementary Table S6. Likelihood-ratio comparisons between the fitted power-law model and alternative distributions

Comparison	Log-likelihood ratio (R)	p-value
Power-law vs Lognormal	-1.34	0.181
Power-law vs Exponential	0.08	0.935
Power-law vs Truncated power-law	-1.62	0.295

Positive R values indicate support for the power-law model, and negative R values indicate support for the alternative model. None of the comparisons was statistically significant ($p > 0.05$), indicating that the power-law model was not clearly preferred over the alternatives tested.

Supplementary Table S7. Sensitivity analysis of hub-gene definitions across alternative degree thresholds

**Threshold**	**Hub_count**	**Hub_percentage**	**Hub_genes**
Degree ≥ 2	21	21.21%	MLH1; TP53; MSH2; CD274; TGFBR2; BRAF; KRAS; PTEN; RNF43; ACVR2A; ARID1A; BRCA2; COX-2; CTNNB1; ERBB2; FHIT; KMT2D; PDCD1; PIK3CA; TYMS; VEGF
Degree ≥ 3	9	9.09%	MLH1; TP53; MSH2; CD274; TGFBR2; BRAF; KRAS; PTEN; RNF43
Degree ≥ 4	6	6.06%	MLH1; TP53; MSH2; CD274; TGFBR2; BRAF
Degree ≥ 5	5	5.05%	MLH1; TP53; MSH2; CD274; TGFBR2

The table lists the number, proportion, and identities of genes classified as hubs under alternative degree thresholds in the revised primary simple gene–cancer network.

Supplementary Table S8. Full enrichment statistics for revised hub genes (degree ≥ 3)

Full over-representation analysis results generated using Enrichr through GSEApy for the revised hub-gene set (n = 9) are provided in the accompanying Excel file, *Supplementary_Table_S8_Full_enrichment_results.xlsx*. Reported fields include the gene-set database, term, overlap, odds ratio, raw p-value, Enrichr-reported adjusted p-value, Benjamini–Hochberg false discovery rate (BH-FDR), combined score, contributing genes, and background mode. Because custom background submission was not supported in the final Enrichr runs, the reported statistics correspond to the default database background.

Supplementary Table S9. Additional descriptive checks using simple gene-level publication-based rankings

Comparison	Genes
Simple hubs (degree ≥ 3)	BRAF, CD274, KRAS, MLH1, MSH2, PTEN, RNF43, TGFBR2, TP53
Top 9 by publication-weighted degree	MLH1, BRAF, TP53, CD274, KRAS, TGFBR2, MSH2, PTEN, COX-2
Top 9 by publication-normalized degree	TP53, MSH2, MLH1, TGFBR2, RNF43, CD274, ACVR2A, ARID1A, BRCA2

The table compares the nine hub genes identified from the primary simple network with the top nine genes ranked by two simple gene-level publication-based summaries: publication-weighted degree and publication-normalized degree. These comparisons were included as additional descriptive checks of literature frequency bias at the gene level and were not used to define the primary network. Publication-weighted degree was defined as the sum of supporting PMIDs across all edges. Publication-normalized degree was defined as simple degree / log2(PMID count + 1).

Supplementary Table S10. Publication-adjusted logistic regression for universal gene status

Variable	Coefficient	OR
Hub_degree_ge_3	0.82	2.28
log2_pubcount_plus1	1.57	4.81

Penalized logistic regression (L2 regularization) was used because complete separation was present in the data. The outcome was universal gene status (degree ≥ 2), and predictors were hub status and log-transformed publication count. Because hub status is definitionally nested within universal status, this model was used only as a supplementary sensitivity analysis and not for primary inference.

Supplementary Table S11. Cohort-specific and pooled mutation validation results for the revised hub genes in selected TCGA cohorts

Cohort	MSI-H (n)	non-MSI-H (n)	Significant genes (FDR < 0.05)
UCEC	166	362	5/9: KRAS, MLH1, PTEN, RNF43, TP53
COADREAD	88	496	8/9: BRAF, KRAS, MLH1, MSH2, PTEN, RNF43, TGFBR2, TP53
STAD	82	356	8/9: BRAF, CD274, KRAS, MLH1, MSH2, PTEN, RNF43, TP53
Pooled	336	1,214	9/9: all hub genes

Supplementary Table S12. Pooled mutation validation results for the revised hub genes across selected TCGA cohorts

Gene	MSIH_mut	MSIH_total	MSIH_rate	Non_MSIH_mut	Non_MSIH_total	Non_MSIH_rate	OR_haldane	CI_lower	CI_upper	FDR	Significance
BRAF	60	336	17.86	40	1214	3.29	6.3454	4.1736	9.6473	0	***
CD274	10	336	2.98	7	1214	0.58	5.1776	2.0124	13.3211	0.001018	**
KRAS	98	336	29.17	259	1214	21.33	1.5207	1.1587	1.9959	0.003348	**
MLH1	42	336	12.5	24	1214	1.98	7.0124	4.1971	11.7161	0	***
MSH2	37	336	11.01	35	1214	2.88	4.1601	2.5841	6.6974	0	***
PTEN	175	336	52.08	229	1214	18.86	4.6664	3.607	6.0369	0	***
RNF43	128	336	38.1	38	1214	3.13	18.8334	12.7633	27.7904	0	***
TGFBR2	24	336	7.14	32	1214	2.64	2.8526	1.6639	4.8902	0.000464	***
TP53	85	336	25.3	635	1214	52.31	0.31	0.2367	0.4061	0	***

Supplementary Table S13. Cohort-specific and pooled expression validation results for the revised hub genes in selected TCGA cohorts

Cohort	MSI-H (n)	non-MSI-H (n)	Significant genes (FDR < 0.05)
UCEC	166	362	5/9: BRAF, MLH1, PTEN, RNF43, TP53
COADREAD	88	496	8/9: BRAF, CD274, KRAS, MLH1, PTEN, RNF43, TGFBR2, TP53
STAD	82	356	8/9: CD274, KRAS, MLH1, MSH2, PTEN, RNF43, TGFBR2, TP53
Pooled	336	1,214	9/9: all hub genes

Supplementary Table S14. Pooled expression validation results for the revised hub genes across selected TCGA cohorts

Gene	MSIH_n_total	Non_MSIH_n_total	Weighted_median_log2FC_summary	Combined_P_value	FDR	Significance
BRAF	331	1187	-0.1898	0.000174	0.000219	***
CD274	331	1187	0.8181	0	0	***
KRAS	331	1187	0.1182	0.000195	0.000219	***
MLH1	331	1187	-2.4317	0	0	***
MSH2	331	1187	0.0199	0.024218	0.024218	*
PTEN	331	1187	-0.0106	4.00E-06	6.00E-06	***
RNF43	331	1187	-0.7679	0	0	***
TGFBR2	331	1187	-0.6354	0	0	***
TP53	331	1187	0.2319	0	0	***
